# Supplementary material for: Activation of Liver X Receptors and Peroxisome Proliferator-Activated Receptors by Lipid Extracts of Brown Seaweeds: A Potential Application in Alzheimer’s Disease?
Source: Nutrients. 2023 Jun 30;15(13):3004. doi: 10.3390/nu15133004 (PMC10347067; doi:10.3390/nu15133004)
Supplement: Supplementary file 1 [file nutrients-15-03004-s001.zip › nutrients-2430289-supplementary.pdf]

Supplementary data

Table S1. The molecular composition and relative content of phospholipid in seaweed extracts.

Sub classes	No.	RT	Molecular species	Acyl-1	Acyl-2	*A. esculenta*	*A. nodosum*	*F. vesiculosis*	*H. elongata*	*S. fusiforme*	*S. latissima*	*S. muticum*
LPA	1	3.41	LPA 16:0	16:0	/	-	-	-	-	-	25.84	59.15
	2	3.69	LPA 18:1	18:1	/	100.00	-	-	-	-	-	-
	3	2.58	LPA 20:4	20:4	/	-	-	-	-	-	74.16	40.85
PA	1	6.61	PA 30:0	16:0	14:0	-	-	0.03	-	-	-	-
	2	6.17	PA 32:2	14:0	18:2	5.23	-	0.04	-	-	-	-
				16:1	16:1							
	3	6.62	PA 32:1	14:0	18:1	8.78	11.89	1.11	-	-	19.29	-
				16:1	16:0							
	4	7.33	PA 32:0	14:0	18:0	1.98	6.73	1.75	-	-	-	-
				16:0	16:0							
	5	5.43	PA 34:5	14:0	20:5	35.53	-	-	-	-	-	-
	6	6.07	PA 34:4	14:0	20:4	24.85	19.46	9.38	9.88	-	41.13	-
				16:0	18:4							
	7	6.32	PA 34:3	16:0	18:3	0.07	-	-	-	-	0.03	-
				14:0	20:3							
	8	6.72	PA 34:2	16:0	18:2	0.10	-	0.11	-	-	-	-
	9	7.74	PA 34:1	16:0	18:1	0.86	0.26	0.02	0.09	-	0.82	-
	10	6.24	PA 36:5	16:0	20:5	0.28	0.41	0.07	-	-	0.96	-
	11	6.61	PA 36:4	16:0	20:4	21.93	16.21	5.40	18.71	-	36.81	37.67
				18:2	18:2							
	12	7.39	PA 36:2	18:1	18:1	-	0.94	5.36	-	-	-	-
	13	5.61	PA 38:8	18:4	20:4	-	6.90	0.11	-	-	-	-
				18:3	20:5							
	14	5.84	PA 38:7	18:3	20:4	-	-	32.23	-	-	-	-
			PA 38:7	18:2	20:5							
	15	6.21	PA 38:6	18:1	20:5	0.38	0.23	35.00	34.38	-	-	-
				18:0	20:4							
	16	6.64	PA 38:5	20:4	18:1	-	4.41	1.19	7.99	-	-	6.51
	17	7.34	PA 38:4	18:0	20:4	-	9.10	0.66	4.46	-	-	7.94
				18:1	20:3							
				18:3	20:1							
	18	5.74	PA 40:9	20:5	20:4	-	23.46	7.55	11.31	-	-	42.03
	19	8.20	PA 40:4	20:0	20:4	-	-	-	2.43	-	0.98	5.85
	20	9.27	PA 42:4	20:4	22:0	-	-	-	10.75	-	-	-
Sub classes	No.	RT	Molecular species	Acyl-1	Acyl-2	*A. esculenta*	*A. nodosum*	*F. vesiculosis*	*H. elongata*	*S. fusiforme*	*S. latissima*	*S. muticum*
LPG	1	2.84	LPG 16:1	16:1	/	29.19	15.73	8.32	28.35	24.13	23.96	25.39
	2	3.14	LPG 16:0	16:0	/	22.79	29.58	54.75	25.12	25.37	37.38	55.10

	3	3.69	LPG 17:0	17:0	/	0.41	-	-	-	-	-	-
	4	1.79	LPG 18:4	18:4	/	0.50	-	-	-	-	-	-
	5	1.29	LPG 18:3	18:3	/	4.24	3.94	0.16	7.11	16.26	-	4.29
	6	2.56	LPG 18:2	18:2	/	4.01	-	-	-	-	-	-
	7	3.39	LPG 18:1	18:1	/	38.37	50.74	36.77	39.42	34.24	38.65	15.22
	8	4.08	LPG 18:0	18:0	/	0.01	-	-	-	-	-	-
	9	3.70	LPG 19:1	19:1	/	0.47	-	-	-	-	-	-
PG	1	6.03	PG 30:1	16:1	14:0	0.72	-	-	-	-	0.13	-
	2	6.14	PG 30:0	16:0	14:0	1.14	-	19.86	-	-	-	-
	3	5.83	PG 32:2	16:1	16:1	1.71	-	1.91	-	-	4.10	8.20
	4	6.27	PG 32:1	16:0	16:1	8.74	12.21	4.07	14.82	17.69	16.55	26.90
	5	6.80	PG 32:0	16:0	16:0	1.93	4.63	12.61	3.44	-	0.80	-
	6	6.54	PG 33:1	18:1	15:0	0.53	-	-	1.55	-	0.91	-
				16:0	17:1							
	7	5.42	PG 34:5	20:5	14:0	-	-	-	2.47	-	-	-
	8	5.62	PG 34:4	16:1	18:3	-	17.41	7.64	0.38	0.50	-	-
	9	6.00	PG 34:3	16:0	18:3	0.10	0.16	0.15	1.06	0.09	0.06	0.43
	10	6.37	PG 34:2	16:0	18:2	13.79	18.34	24.68	15.83	63.78	22.46	25.05
	11	6.83	PG 34:1	16:0	18:1	51.97	25.53	10.46	55.21	13.33	51.58	23.08
	12	7.66	PG 34:0	16:0	18:0	-	-	11.48	-	-	-	-
	13	7.18	PG 35:1	18:1	17:0	1.09	-	-	0.60	-	-	-
				16:0	19:1							
	14	5.18	PG 36:7	18:3	18:4	2.78	-	-	-	-	-	0.20
	15	5.67	PG 36:5	16:0	20:5	0.03	-	-	-	-	-	-
				20:4	16:1							
				18:1	18:4		-	-	-	-	-	-
				18:2	18:3							
	16	6.04	PG 36:4	18:1	18:3	1.43	-	-	-	0.36	0.30	0.27
				18:2	18:2							
				16:0	20:4							
	17	6.39	PG 36:3	18:2	18:1	4.77	-	-	-	-	-	-
	18	6.84	PG 36:2	18:2	18:0	4.52	18.25	0.80	2.00	4.25	2.28	3.66
				18:1	18:1							
	19	7.58	PG 36:1	18:0	18:1	4.75	3.48	6.31	1.76	-	0.83	1.95
				20:1	16:0							
	20	7.33	PG 37:2	18:1	19:1	-	-	0.03	0.87	-	-	-
	21	8.42	PG 38:1	16:0	22:1	-	-	-	-	-	-	10.26
Sub classes	No.	RT	Molecular species	Acyl-1	Acyl-2	*A. esculenta*	*A. nodosum*	*F. vesiculosis*	*H. elongata*	*S. fusiforme*	*S. latissima*	*S. muticum*
LPE	1	2.73	LPE 20:4	20:4	/	-	-	-	-	-	-	100.00
PE	1	6.23	PE 32:2	16:1	16:1	-	-	-	-	-	-	13.33
	2	6.77	PE 32:1	16:1	16:0	57.77	46.23	-	10.26	-	9.78	33.43

	3	6.37	PE 36:5	16:0	20:5	-	-	-	-	-	2.59	1.33
	4	6.75	PE 36:4	16:0	20:4	42.23	-	-	14.85	-	29.12	7.96
	5	7.50	PE 36:2	18:1	18:1	-	-	100.00	-	-	4.17	-
	6	7.48	PE 38:4	18:0	20:4	-	-	-	7.39	-	4.30	-
	7	5.86	PE 40:9	20:5	20:4	-	-	-	9.87	-	9.22	6.13
	8	6.21	PE 40:8	20:4	20:4	-	43.23	-	34.51	-	34.70	23.73
	9	7.51	PE 40:5	20:4	20:1	-	-	-	-	-	-	4.62
	10	8.36	PE 40:4	20:4	20:0	-	-	-	7.92	-	6.11	-
	11	8.69	PE 42:5	20:5	22:0	-	-	-	0.03	-	-	4.74
	12	9.40	PE 42:4	20:4	22:0	-	10.55	-	12.27	-	-	4.72
	13	10.68	PE 44:4	20:4	24:0	-	-	-	2.89	-	-	-
Sub classes	No.	RT	Molecular species	Acyl-1	Acyl-2	*A. esculenta*	*A. nodosum*	*F. vesiculosis*	*H. elongata*	*S. fusiforme*	*S. latissima*	*S. muticum*
LPI	1	2.72	LPI 16:0	16:0	/	-	17.19	-	-	-	-	-
	2	2.30	LPI 18:2	18:2	/	-	21.32	-	-	-	-	-
	3	3.06	LPI 18:1	18:1	/	100.00	61.49	-	-	-	-	-
PI	1	6.07	PI 30:0	16:0	14:0	-	-	-	-	-	1.09	-
			PI 30:0	15:0	15:0							
			PI 30:0	14:0	16:0							
	2	6.12	PI 32:1	14:0	18:1	4.35	4.76	0.39	6.28	9.55	20.84	-
	3	5.89	PI 34:3	18:3	16:0	-	-	-	6.56	-	-	-
	4	6.23	PI 34:2	16:0	18:2	7.20	19.44	9.34	40.64	33.86	11.41	-
				16:1	18:1							
	5	6.66	PI 34:1	16:0	18:1	85.33	54.47	6.92	46.52	38.77	64.93	-
	6	6.13	PI 36:4	16:0	20:4	-	2.71	25.74	-	17.82	1.18	-
	7	6.27	PI 36:3	18:1	18:2	-	0.01	43.61	-	-	-	-
	8	6.72	PI 36:2	18:1	18:1	1.03	15.16	13.99	-	-	-	-
	9	7.35	PI 36:1	18:1	18:0	2.09	3.45	0.01	-	-	0.54	-
Sub classes	No.	RT	Molecular species	Acyl-1	Acyl-2	*A. esculenta*	*A. nodosum*	*F. vesiculosis*	*H. elongata*	*S. fusiforme*	*S. latissima*	*S. muticum*
PS	1	5.85	PS 34:1	16:0	18:1	-	-	-	-	-	-	100.00
Sub classes	No.	RT	Molecular species	Acyl-1	Acyl-2	*A. esculenta*	*A. nodosum*	*F. vesiculosis*	*H. elongata*	*S. fusiforme*	*S. latissima*	*S. muticum*
LPC	1	2.38	LPC 14:0	14:0	/	-	-	-	-	-	7.45	-
	2	2.50	LPC 16:1	16:1	/	-	-	-	-	-	0.17	-
	3	3.46	LPC 16:0	16:0	/	4.28	-	-	-	-	7.24	-
	4	3.96	LPC 17:0	17:0	/	6.38	100.00	-	100.00	-	55.40	100.00
	5	2.23	LPC 18:3	18:3	/	3.67	-	-	-	-	-	-
	6	2.86	LPC 18:2	18:2	/	14.87	-	-	-	-	4.87	-
	7	3.65	LPC 18:1	18:1	/	19.05	-	-	-	-	4.91	-
	8	1.32	LPC 20:5	20:5	/	10.58	-	-	-	-	4.04	-
	9	2.72	LPC 20:4	20:4	/	34.68	-	-	-	-	15.92	-
	10	3.16	LPC 20:3	20:3	/	5.84	-	-	-	-	-	-
	11	3.87	LPC 20:2	20:2	/	0.64	-	-	-	-	-	-

	1	5.96	PC 28:0	14:0	14:0	0.41	-	-	-	-	-	-
	2	6.03	PC 30:1	16:1	14:0	0.76	-	-	-	-	6.41	-
	3	6.53	PC 30:0	14:0	16:0	0.38	-	-	-	-	1.12	-
	4	6.28	PC 31:1	17:1	14:0	0.48	-	-	-	-	4.20	-
				16:1	15:0							
				18:1	13:0							
	5	5.51	PC 32:4	14:0	18:4	1.43	-	-	-	-	0.65	-
	6	5.79	PC 32:3	18:3	14:0	2.18	-	-	-	-	2.08	-
	7	6.13	PC 32:2	18:2	14:0	6.89	-	-	-	-	9.82	-
	8	6.54	PC 32:1	18:1	14:0	7.20	-	-	-	-	10.33	-
				16:1	16:0							
	10	5.71	PC 34:5	20:5	14:0	5.84	-	-	-	-	4.02	-
	11	6.03	PC 34:4	20:4	14:0	11.45	-	-	-	-	13.35	-
				18:4	16:0							
	12	6.30	PC 34:3	20:3	14:0	3.71	-	-	-	-	3.16	-
				18:3	16:0							
	13	6.73	PC 34:2	18:2	16:0	6.85	-	-	-	-	5.44	-
				20:2	14:0							
	14	7.24	PC 34:1	18:1	16:0	6.92	-	-	-	-	4.82	-
				20:1	14:0							
PC	15	7.98	PC 34:0	17:0	17:0	3.08	80.06	95.52	99.84	100.00	7.03	99.98
	14	6.30	PC 35:4	20:4	15:0	-	-	-	-	-	0.30	-
	16	6.05	PC 36:5	20:5	16:0	1.38	-	-	-	-	4.13	-
				20:4	16:1	6.49						
				18:1	18:4							
	17	6.55	PC 36:4	20:4	16:0	5.72	-	-	-	-	6.78	-
				18:1	18:3	1.31						
	18	7.23	PC 36:2	18:1	18:1	0.99	-	0.13	-	-	1.19	-
				18:2	18:0							
				16:0	20:2							
	19	8.68	PC 38:9	20:5	18:4	0.03	19.94	4.35	0.16	-	-	0.02
	20	5.82	PC 38:7	18:3	20:4	3.97	-	-	-	-	1.32	-
				18:2	20:5							
	21	6.17	PC 38:6	18:2	20:4	6.94	-	-	-	-	3.19	-
				18:1	20:5							
	22	6.56	PC 38:5	18:1	20:4	4.23	-	-	-	-	1.51	-
				18:0	20:5							
	23	5.43	PC 40:10	20:5	20:5	2.00	-	-	-	-	-	-
				20:4	20:5							
	21	5.73	PC 40:9	20:4	20:5	-	-	-	-	-	3.41	-
	24	6.05	PC 40:8	20:4	20:4	8.54	-	-	-	-	5.72	-

	25	6.29	PC 40:7	20:3	20:4	0.84	-	-	-	-	-	-

RT, Retention Time (in minutes); LPA, Lysophosphatidic Acid; PA, Phosphatidic Acid; LPG, Lysophosphatidylglycerol; PG, Phosphatidylglycerol; LPE, Lysophosphatidylethanolamine; PE, Phosphatidylethanolamine; LPI, Lysophosphatidylinositol; PI, Phosphatidylinositol; PS, Phosphatidylserine; LPC, Lysophosphatidylcholine; PC, Phosphatidylcholine.

Table S2. The molecular composition and relative content of glyceroglycolipid, diglyceride and triglyceride in seaweed extracts.

Sub classes	No.	RT	Molecular species	Acyl-1	Acyl-2	Acyl-3	*A. esculenta*	*A. nodosum*	*F. vesiculosis*	*H. elongata*	*S. fusiforme*	*S. latissima*	*S. muticum*
MGMG	1	2.95	MGMG 14:0	C14:0	/	/	-	-	-	-	-	0.01	0.29
	2	3.59	MGMG 15:0	C15:0	/	/	9.74	0.42	0.72	1.04	1.93	8.12	0.02
	3	3.21	MGMG 16:1	C16:1	/	/	0.45	0.64	0.49	1.21	2.80	0.38	0.09
	4	4.29	MGMG 16:0	C16:0	/	/	22.32	15.91	44.11	51.78	43.82	80.89	0.57
	6	1.99	MGMG 18:4	C18:4	/	/	42.10	33.71	16.10	16.38	13.45	2.22	1.21
	7	2.83	MGMG 18:3	C18:3	/	/	4.52	11.45	9.48	12.62	7.38	0.70	96.82
	8	3.56	MGMG 18:2	C18:2	/	/	-	0.31	-	0.01	0.07	-	-
	9	4.51	MGMG 18:1	C18:1	/	/	18.20	26.49	25.09	15.58	27.10	6.97	0.26
	10	3.21	MGMG 20:4	C20:4	/	/	2.61	9.27	3.84	1.19	3.30	0.71	0.24
	11	4.10	MGMG 20:3	C20:3	/	/	0.06	1.80	0.17	0.19	0.15	-	0.50
Sub classes	No.	RT	Molecular species	Acyl-1	Acyl-2	Acyl-3	*A. esculenta*	*A. nodosum*	*F. vesiculosis*	*H. elongata*	*S. fusiforme*	*S. latissima*	*S. muticum*
MGDG	1	7.61	MGDG 28:1	C14:0	C14:1	/	0.01	-	-	-	0.01	0.03	-
	2	8.27	MGDG 30:1	C14:0	C16:1	/	0.97	0.31	0.65	1.06	3.18	16.91	0.85
	3	9.05	MGDG 30:0	C16:0	C14:0	/	0.23	0.15	0.40	0.51	0.95	0.02	0.31
	4	7.57	MGDG 32:4	C14:0	C18:4	/	0.01	1.13	0.21	1.22	0.92	0.09	0.29
	5	7.93	MGDG 32:3	C16:1	C16:2	/	0.92	0.61	2.66	0.59	1.23	5.54	0.97
				C14:0	C18:3	/							
	6	8.37	MGDG 32:2	C16:1	C16:1	/	2.71	0.24	0.41	0.08	0.75	20.40	0.51
	7	8.53	MGDG 32:2	C14:0	C18:2	/	1.49	1.15	3.26	4.77	1.80	6.16	2.17
	8	9.17	MGDG 32:1	C16:0	C16:1	/	8.88	2.84	7.72	12.70	12.12	0.52	1.04
				C14:0	C18:1	/							
				C18:1	C14:0	/							
	9	8.82	MGDG 33:2	C15:0	C18:2	/	44.31	1.01	2.21	3.81	3.10	4.97	0.39
	10	9.59	MGDG 33:1	C15:0	C18:1	/	0.01	0.06	-	-	0.01	0.11	0.40
				C16:0	C17:1	/							
			MGDG 34:8	C18:4	C16:4	/							
	11	7.15	MGDG 34:6	C14:1	C20:5	/	0.22	0.47	0.34	0.47	0.54	0.61	0.36
	12	7.63	MGDG 34:5	C16:1	C18:4	/	1.90	1.59	1.51	0.77	1.24	1.58	10.99
	13	8.27	MGDG 34:4	C16:0	C18:4	/	3.52	0.95	1.37	0.56	1.64	5.20	0.93
				C14:0	C20:4	/					0.00		0.00

	14	8.54	MGDG 34:3	C16:1	C18:2	/	0.01	0.03	0.23	0.29	0.05	0.67	2.07
	15	8.7/7.05	MGDG 34:3	C16:0	C18:3	/	2.32	2.09	4.77	5.00	6.46	17.75	2.63
	16	9.21	MGDG 34:2	C18:1	C16:1	/	2.97	1.86	3.44	19.23	9.26	1.31	53.13
				C16:1	C18:1	/							
				C16:0	C18:2	/							
	17	10.03	MGDG 34:1	C16:0	C18:1	/	7.24	3.94	5.32	23.08	21.01	0.14	0.33
				C18:1	C16:0	/							
	18	7.28	MGDG 36:7	C18:3	C18:4	/	6.96	7.33	4.45	12.46	8.95	5.44	2.30
	19	7.61	MGDG 36:6	C18:3	C18:3	/	1.72	3.99	3.91	7.80	6.24	1.63	7.96
	20	8.48	MGDG 36:5	C16:0	C20:5	/	0.30	1.62	1.51	0.26	0.70	0.27	2.61
	21	8.14	MGDG 36:5	C18:2	C18:3	/	1.50	1.52	1.89	0.21	0.86	0.55	0.02
	22			C18:1	C18:4	/							
	23	8.78	MGDG 36:4	C18:2	C18:2	/	2.71	8.08	12.39	1.77	4.92	3.21	0.45
				C18:1	C18:3	/							
				C16:0	C20:4	/							
	24	9.39	MGDG 36:3	C18:1	C18:2	/	0.34	0.75	0.97	0.09	0.37	1.95	0.16
		9.56		C16:0	C20:3	/	0.38	1.31	1.86	0.55	0.68	0.37	3.49
	25	10.22	MGDG 36:2	C18:1	C18:1	/	0.28	2.17	2.52	0.54	0.84	-	3.59
				C16:0	C20:2	/							
	26	10.36	MGDG 36:1	C20:1	C16:0	/	0.42	0.29	0.83	0.37	1.00	0.10	0.06
				C18:0	C18:1	/							
	27	7.97	MGDG 38:7	C18:3	C20:4	/	0.96	13.81	7.04	0.54	2.02	1.01	0.01
	28	8.39	MGDG 38:6	C18:2	C20:4	/	1.69	22.82	14.78	0.48	1.91	2.62	0.47
				C18:3	C20:3	/							
	29	9.13	MGDG 38:5	C18:1	C20:4	/	0.85	8.60	9.03	0.10	0.99	0.16	0.16
		8.91		C18:2	C20:3	/	1.09	5.70	2.04	0.14	1.80	0.53	0.06
	30	12.89	MGDG 38:1	C18:1	C20:0	/	0.01	0.04	0.01	0.03	-	0.06	1.27
	31			C16:0	C22:1	/							
	32	7.41	MGDG 40:8	C20:4	C20:4	/	3.05	3.55	2.24	0.49	4.44	0.09	0.01
Sub classes	No.	RT	Molecular species	Acyl-1	Acyl-2	Acyl-3	*A. esculenta*	*A. nodosum*	*F. vesiculosis*	*H. elongata*	*S. fusiforme*	*S. latissima*	*S. muticum*
DGDG	1	7.61	DGDG 28:0	C14:0	C14:0	/	0.10	0.37	0.33	0.06	0.07	0.60	3.11
	2	7.69	DGDG 30:1	C14:0	C16:1	/	0.27	1.18	0.42	1.38	1.15	11.51	1.16
	3	8.33	DGDG 30:0	C16:0	C14:0	/	0.20	0.79	1.29	0.22	0.58	0.07	0.31
	4	8.06	DGDG 31:1	C15:0	C16:1	/	0.02	0.26	0.10	0.04	0.06	-	0.09
				C14:0	C17:1	/							
				C13:0	C18:1	/							
	5	7.34	DGDG 32:3	C14:0	C18:3	/	1.82	0.98	1.38	1.40	1.15	4.94	42.29

	6	7.83	DGDG 32:2	C14:0	C18:2	/	8.70	4.72	3.76	6.15	3.33	13.62	0.18
	7	9.16	DGDG 32:0	C16:0	C16:0	/	0.05	0.25	0.33	0.12	0.22	0.01	-
	8	7.18	DGDG 34:5	C14:0	C20:5	/	0.43	0.97	1.10	0.32	1.17	0.40	1.48
	9	7.67	DGDG 34:4	C14:0	C20:4	/	0.81	0.24	0.50	0.44	0.52	0.39	0.30
				C16:0	C18:4	/							
	10	8.66	DGDG 33:2	C16:0	C18:2	/	3.66	3.75	3.93	18.04	11.22	0.03	0.27
	11	9.29	DGDG 33:1	C16:0	C18:1	/	15.25	16.43	18.67	19.79	15.78	14.51	3.16
	12	6.35	DGDG 36:8	C18:4	C18:4	/	0.04	0.01	0.04	0.25	0.10	0.10	0.45
	13	7.08	DGDG 36:6	C18:3	C18:3	/	0.91	1.14	1.34	5.15	5.70	0.59	-
	14	7.24	DGDG 36:6	C16:1	C20:5	/	0.51	3.03	2.31	0.51	1.83	1.89	3.72
				C18:2	C18:4	/							
	15	8.09	DGDG 36:4	C18:2	C18:2	/	0.87	2.15	1.95	0.60	1.12	0.21	0.09
				C18:1	C18:3	/							
	16	8.41	DGDG 36:4	C16:0	C20:4	/	0.08	0.62	1.37	0.02	0.36	0.01	-
	17	8.63	DGDG 36:3	C18:1	C18:2	/	0.07	0.01	0.04	-	-	0.16	7.22
	18	9.25	DGDG 36:2	C18:1	C18:1	/	0.50	3.42	1.49	0.36	0.56	0.01	20.80
	19	10.18	DGDG 36:1	C18:0	C18:1	/	0.24	0.92	1.10	0.17	0.41	-	-
	20	6.59	DGDG 38:9	C18:4	C20:5	/	47.87	18.19	14.21	23.50	25.80	39.26	1.10
	21	7.05	DGDG 38:8	C18:3	C20:5	/	9.58	22.31	21.06	17.25	26.95	9.05	0.97
	22	7.95	DGDG 38:6	C18:1	C20:5	/	1.18	12.74	15.17	0.06	0.45	0.52	1.40
	23	8.45	DGDG 38:5	C18:1	C20:4	/	6.07	4.49	7.81	4.14	1.16	1.99	11.90
	24	8.90	DGDG 38:4	C18:1	C20:3	/	0.76	1.04	0.28	0.02	0.31	0.13	-
Sub classes	No.	RT	Molecular species	Acyl-1	Acyl-2	Acyl-3	*A. esculenta*	*A. nodosum*	*F. vesiculosis*	*H. elongata*	*S. fusiforme*	*S. latissima*	*S. muticum*
SQDG	1	4.78	SQDG 24:0	C16:0	C8:0	/	0.56	-	0.04	0.13	-	-	0.06
	2	5.14	SQDG 26:0	C14:0	C12:0	/	-	-	-	-	-	-	-
	3	3.38	SQDG 28:6	C12:6	C16:0	/	-	-	-	-	-	-	0.03
	4	5.21	SQDG 28:1	C14:0	C14:1	/	-	-	-	-	-	0.01	-
	5	5.65	SQDG 28:0	C14:0	C14:0	/	2.92	-	-	0.01	-	-	-
	6	5.74	SQDG 29:0	C14:0	C15:0	/	-	-	-	-	-	0.90	-
	7	5.56	SQDG 30:1	C14:0	C16:1	/	-	1.08	0.61	0.68	2.47	2.35	0.79
	8	5.88	SQDG 30:0	C16:0	C14:0	/	45.08	55.14	69.75	26.61	-	55.39	42.80
	9	4.42	SQDG 31:5	C16:0	C15:5	/	-	-	-	-	-	-	-
	10	5.81	SQDG 31:1	C14:0	C17:1	/	-	-	0.19	-	-	0.22	0.15
	11	4.48	SQDG 31:0	C14:0	C17:0	/	0.41	1.00	-	0.82	2.71	0.32	0.80
	12	4.57	SQDG 32:5	C16:0	C16:5	/	-	-	-	-	-	0.01	-
	13	5.18	SQDG 32:4	C14:0	C18:4	/	1.96	-	0.19	-	-	-	-
	14	5.35	SQDG 32:3	C14:0	C18:3	/	4.13	2.07	1.25	0.89	5.24	2.21	-

	15	5.67	SQDG 32:2	C14:0	C18:2	/	-	-	-	-	-	-	-
	16	5.99	SQDG 32:1	C14:0	C18:1	/	3.71	8.48	6.45	6.31	16.99	-	10.00
	17	6.43	SQDG 32:0	C16:0	C16:0	/	2.01	3.13	4.03	2.18	7.54	2.57	4.21
	18	5.59	SQDG 33:3	C18:3	C15:0	/	0.01	1.74	1.19	-	-	-	-
	19	5.93	SQDG 33:2	C15:0	C18:2	/	-	-	1.01	-	-	1.04	-
	20	6.27	SQDG 33:1	C18:1	C15:0	/	-	-	-	0.02	0.13	0.04	0.03
	21	4.79	SQDG 33:0	C14:0	C19:0	/	-	-	-	0.04	0.07	-	0.02
	22	5.44	SQDG 34:4	C16:1	C18:3	/	0.26	0.12	0.06	-	-	-	0.16
	23	5.76	SQDG 34:3	C18:3	C16:0	/	-	0.01	-	0.01	-	-	0.06
	24	6.29	SQDG 34:2	C18:2	C16:0	/	0.01	-	-	-	-	-	-
	25	6.41	SQDG 34:1	C18:1	C16:0	/	34.88	15.41	13.70	40.70	-	32.57	25.26
	26	7.24	SQDG 34:0	C18:0	C16:0	/	-	-	-	-	1.78	-	0.46
	27	4.85	SQDG 35:3	C19:3	C16:0	/	0.02	-	-	0.04	-	-	0.02
	28	5.17	SQDG 36:9	C16:0	C20:9	/	-	-	-	-	-	-	0.01
	29	6.90	SQDG 35:1	C17:0	C18:1	/	-	-	-	9.92	-	-	-
	30	5.17	SQDG 35:0	C19:0	C16:0	/	-	0.02	-	-	-	-	-
	31	5.27	SQDG 36:6	C18:3	C18:3	/	-	0.24	-	-	0.99	-	-
	32	5.71	SQDG 36:5	C16:0	C20:5	/	2.93	2.55	0.84	3.64	-	1.51	2.70
	33	5.85	SQDG 36:4	C18:1	C18:3	/	0.29	0.10	0.09	0.22	1.80	0.20	-
	34	6.18	SQDG 36:3	C18:1	C18:2	/	0.79	2.17	0.56	-	-	-	0.57
	35	6.61	SQDG 36:2	C18:1	C18:1	/	0.02	-	-	-	0.01	-	-
	36	7.15	SQDG 36:1	C20:1	C16:0	/	-	-	-	-	30.82	0.55	11.87
	37	8.07	SQDG 36:0	C20:0	C16:0	/	-	-	-	-	0.25	0.09	-
	38	4.19	SQDG 37:5	C19:2	C18:3	/	-	-	-	-	-	-	-
	39	4.96	SQDG 38:9	C18:4	C20:5	/	0.01	-	-	-	-	0.01	-
	40	5.19	SQDG 38:8	C18:3	C20:5	/	-	0.36	-	-	2.00	-	-
	41	8.41	SQDG 38:7	C15:0	C23:7	/	-	0.16	-	-	-	-	-
	42	5.76	SQDG 38:6	C20:4	C18:2	/	-	-	-	-	0.43	-	-
	43	7.91	SQDG 38:1	C22:1	C16:0	/	-	-	-	-	26.49	-	-
	44	8.88	SQDG 38:0	C22:0	C16:0	/	-	-	-	0.01	0.03	-	-
	45	6.02	SQDG 39:1	C23:1	C16:0	/	-	-	-	-	0.19	-	-
	46	5.95	SQDG 39:0	C23:0	C16:0	/	-	-	-	-	0.05	-	-
	47	8.74	SQDG 40:1	C24:1	C16:0	/	-	6.17	-	0.06	-	-	-
	48	10.25	SQDG 40:0	C24:0	C16:0	/	-	-	-	7.70	-	-	-
	49	6.47	SQDG 41:0	C16:0	C25:0	/	-	-	0.01	-	-	-	-
	50	9.07	SQDG 42:2	C18:1	C24:1	/	-	0.02	-	-	-	-	-
Sub classes	No.	RT	Molecular species	Acyl-1	Acyl-2	Acyl-3	*A. esculenta*	*A. nodosum*	*F. vesiculosis*	*H. elongata*	*S. fusiforme*	*S. latissima*	*S. muticum*

	1	9.45	DG 30:1	C14:0	C16:1	/	0.10	0.54	1.52	4.28	1.58	0.32	2.96
	2	8.23	DG 32:2	C14:0	C18:2	/	-	0.00	0.01	0.00	0.01	36.08	-
	3	24.58	DG 32:1	C14:0	C18:1	/	0.28	0.17	0.21	0.42	0.12	9.39	-
	4	9.90	DG 35:4	C15:0	C20:4	/	2.79	0.33	1.25	1.95	1.92	0.58	5.06
	5	10.50	DG 36:4	C16:0	C20:4	/	82.32	6.29	13.90	74.70	68.90	0.94	0.48
	6	13.60	DG 36:1	C18:0	C18:1	/	5.13	5.44	32.29	6.53	8.14	22.14	0.31
	7	10.03	DG 37:5	C17:1	C20:4	/	0.56	1.37	2.11	0.23	0.43	0.04	0.10
DG	8	11.52	DG 37:3	C19:1	C18:2	/	0.02	0.94	1.03	0.18	0.11	-	0.16
	9	14.55	DG 37:0	C18:1	C19:0	/	0.05	0.30	3.57	0.10	0.12	-	0.00
	10	11.22	DG 38:3	C18:1	C20:3	/	5.10	76.56	40.26	7.69	6.13	22.55	32.88
	11	14.79	DG 38:1	C16:0	C22:1	/	-	-	-	0.01	0.01	-	48.54
	12	10.76	DG 40:6	C20:2	C20:4	/	2.34	6.66	1.81	0.49	3.20	1.93	8.35
	13	11.94	DG 40:5	C20:1	C20:4	/	1.26	0.44	1.18	0.08	7.56	0.27	0.95
	14	10.93	DG 42:7	C20:3	C22:4	/	0.02	0.80	0.42	0.49	0.10	3.20	-
	15	14.14	DG 42:5	C20:5	C22:0	/	0.04	0.16	0.45	2.84	1.69	2.56	0.20
Sub classes	No.	RT	Molecular species	Acyl-1	Acyl-2	Acyl-3	*A. esculenta*	*A. nodosum*	*F. vesiculosis*	*H. elongata*	*S. fusiforme*	*S. latissima*	*S. muticum*
	1	20.32	TG 42:0	C12:0	C14:0	C16:0	0.16	0.13	1.19	0.33	0.03	0.05	-
	2	20.80	TG 43:0	C13:0	C15:0	C15:0	0.04	-	0.09	0.03	-	-	-
	3	7.51	TG 44:0	C14:0	C14:0	C16:0	-	-	-	0.09	0.35	0.56	0.03
	4	19.88	TG 45:2	C13:0	C15:0	C17:2	0.11	0.07	0.32	0.19	0.02	0.01	0.03
	5	20.84	TG 45:1	C13:0	C15:0	C17:1	0.25	0.11	1.31	0.39	0.06	0.03	-
	6	18.10	TG 46:4	C14:0	C14:0	C18:4	0.07	0.19	0.76	0.26	0.03	0.04	0.01
				C14:0	C14:1	C18:3	-						
	7	7.89	TG 46:1	C13:0	C16:0	C17:1	1.23	0.01	0.04	0.98	6.46	17.77	1.16
	8	21.70	TG 46:0	C13:0	C16:0	C17:0	1.66	0.02	0.05	1.09	1.11	0.64	1.12
	9	11.74	TG 47:1	C14:0	C16:0	C17:1	0.14	-	-	0.11	0.53	0.66	0.09
TG	10	14.73	TG 48:3	C14:0	C16:0	C18:3	0.42	2.03	4.81	9.46	1.16	0.09	0.10
	11	22.09	TG 48:2	C14:0	C16:1	C18:1	0.04	2.44	0.05	0.06	0.02	-	-
	12	15.15	TG 48:1	C14:0	C16:0	C18:1	23.68	0.24	0.63	9.49	41.15	28.01	41.51
	13	20.32	TG 48:0	C14:0	C16:0	C18:0	2.63	0.02	0.05	0.56	0.48	0.40	0.02
	14	20.76	TG 49:4	C14:0	C15:0	C20:4	0.05	0.02	0.26	0.12	0.03	-	-
	15	11.89	TG 49:2	C14:0	C17:1	C18:1	0.24	-	-	0.02	0.08	0.88	0.08
	16	18.31	TG 50:6	C14:0	C18:3	C18:3	0.49	1.07	0.71	12.52	0.37	0.07	0.42
	17	9.45	TG 50:3	C14:0	C18:1	C18:2	4.74	0.06	0.11	0.64	10.83	21.31	5.18
	18	21.55	TG 51:1	C16:0	C17:0	C18:1	0.41	0.01	-	0.03	0.11	0.07	-
	19	21.57	TG 52:5	C14:0	C18:1	C20:4	8.53	30.74	6.55	6.46	2.17	0.16	-
	20	19.72	TG 52:2	C16:0	C18:1	C18:1	0.03	-	0.02	0.02	0.02	0.15	28.76

	21	9.84	TG 53:2	C16:0	C18:1	C19:1	-	-	0.03	11.53	-	-	0.19
	22	14.75	TG 54:5	C16:0	C18:1	C20:4	30.58	0.32	0.70	3.86	18.72	23.28	19.87
	23	20.29	TG 56:5	C18:0	C18:1	C20:4	2.64	0.01	0.12	0.11	0.40	4.97	0.24
	24	20.52	TG 58:10	C18:1	C20:4	C20:5	9.32	25.50	29.79	11.75	5.73	0.36	0.47
	25	21.43	TG 58:9	C18:0	C20:4	C20:5	11.59	37.02	52.14	8.63	8.82	0.45	0.12
	26	15.48	TG 58:5	C18:1	C20:0	C20:4	0.45	-	0.25	14.25	-	0.01	0.60
	27	12.21	TG 58:3	C18:1	C18:1	C22:1	-	-	-	-	1.33	-	-
	28	18.97<br>16.01	TG 60:6	C18:1<br>C18:1	C20:5<br>C20:4	C22:0<br>C22:1	0.51	-	0.02	7.03	-	-	0.01

RT, Retention Time (in minutes); MGMG, Monogalactosylmonoacylglycerol; MGDG, Monogalactosyldiacylglycerol; DGDG, Digalactosyldiacylglycerol; SQDG, Sulfoquinovosyl Diacylglycerol; DG, Diacylglycerol; TG, Triacylglycerol.

Table S3. The molecular composition and relative content of fatty acids in seaweed extracts.

	A. esculenta	*A. nodosum*	*F. vesiculosis*	*H. elongata*	*S. fusiforme*	*S. latissima*	*S. muticum*
C10:0	N.D	N.D	N.D	N.D	N.D	N.D	N.D
C11:0	N.D	N.D	N.D	N.D	N.D	N.D	N.D
C12:0	0.03	0.07	0.07	0.03	N.D	0.03	0.03
C13:0	0.09	0.02	0.05	N.D	N.D	0.03	N.D
C14:0	8.51	10.23	12.29	7.74	5.55	15.48	4.72
C15:0	0.50	0.30	0.64	0.41	0.56	0.79	N.D
C16:0	21.92	11.12	15.50	33.25	28.15	30.95	35.10
C17:0	0.24	0.16	0.35	0.17	0.10	0.27	0.14
C18:0	2.03	0.78	2.67	0.98	0.58	1.19	0.80
C20:0	0.40	0.19	0.91	0.49	0.28	0.63	0.31
C21:0	N.D	N.D	N.D	N.D	N.D	N.D	N.D
C22:0	N.D	0.13	0.43	1.16	0.94	N.D	0.79
C23:0	N.D	N.D	N.D	N.D	N.D	N.D	N.D
C24:0	N.D	0.15	0.58	0.82	0.26	N.D	0.27
SFA	33.73	23.14	33.48	45.04	36.42	49.38	42.16
C14:1 n-5	N.D	0.36	0.19	0.07	0.07	0.17	0.09
C15:1 n-5	N.D	N.D	N.D	N.D	N.D	N.D	0.54
C16:1 n-7	1.11	1.89	1.45	2.18	4.81	6.88	6.48
C17:1 n-7	N.D	0.42	0.45	0.28	0.21	N.D	0.34
C18:1 n-9	14.51	35.95	34.55	18.75	11.61	18.25	12.46
C18:1 n-7	0.85	0.17	0.24	0.16	0.28	1.06	0.56
C20:1 n-11	0.28	0.15	0.45	N.D	2.38	N.D	3.64
C20:1 n-9	N.D	N.D	N.D	N.D	N.D	N.D	N.D
C20:1 n-7	N.D	N.D	N.D	N.D	N.D	N.D	N.D
C22:1 n-11	N.D	0.18	0.52	N.D	4.14	N.D	4.23
C22:1 n-9	N.D	N.D	N.D	N.D	N.D	N.D	N.D
C24:1 n-9	N.D	0.78	0.96	N.D	N.D	N.D	0.35
MUFA	16.75	39.91	38.80	21.44	23.50	26.35	28.71

C16:4 n-1	N.D	N.D	N.D	N.D	N.D	N.D	0.20
C18:2 n-6	5.74	8.56	7.31	8.28	5.04	4.80	6.25
C18:3 n-6	N.D	N.D	N.D	N.D	N.D	N.D	N.D
C18:3 n-3	9.25	3.88	4.09	7.54	8.72	2.23	3.49
C18:4 n-3	12.33	2.30	1.56	3.27	3.68	3.21	2.07
C20:2 n-6	0.46	2.25	0.50	N.D	0.20	N.D	0.22
C20:3 n-6	0.48	0.72	0.51	0.69	0.37	N.D	0.89
C20:4 n-6	13.10	12.23	9.18	8.61	16.08	9.79	11.40
C20:3 n-3	N.D	0.51	0.18	N.D	N.D	4.25	N.D
C20:4 n-3	0.81	0.96	0.19	0.49	0.46	N.D	0.49
C20:5 n-3	7.36	5.54	4.10	4.64	5.53	N.D	4.11
C22:2 n-6	N.D	N.D	N.D	N.D	N.D	N.D	N.D
C21:5 n-3	N.D	N.D	N.D	N.D	N.D	N.D	N.D
C21:5 n-6	N.D	N.D	N.D	N.D	N.D	N.D	N.D
C22:5 n-3	N.D	N.D	N.D	N.D	N.D	N.D	N.D
C22:6 n-3	N.D	N.D	N.D	N.D	N.D	N.D	N.D
PUFA	49.53	36.95	27.62	33.52	40.08	24.27	29.13
n-6 PUFA	19.78	23.77	17.50	17.58	21.69	14.59	18.76
n-3 PUFA	29.75	13.18	10.12	15.94	18.39	9.69	10.16
n-6/n-3	0.66	1.80	1.73	1.10	1.18	1.51	1.85
PUFA/SFA	1.47	1.60	0.83	0.74	1.10	0.49	0.69
PUFA:MUFA:SFA	1:0.34:0.68	1:1.08:0.63	1:1.41:1.21	1:0.64:1.34	1:0.59:0.91	1:1.09:2.03	1:0.99:1.45
IA	0.85	0.68	0.97	1.17	0.79	1.83	0.93
IT	0.30	0.31	0.52	0.62	0.44	0.95	0.74
UI	200.08	166.52	133.10	135.36	169.79	107.37	132.00

FA, Fatty Acids; SFA, Saturated Fatty Acid; MUFA, Monounsaturated Fatty Acid; PUFA, Polyunsaturated Fatty Acid; IA, Index of Atherogenicity; IT, Index of Thrombogenicity; UI, Unsaturation Index.
